# Comparison of brain activity metrics in Chinese and Russian students while perceiving information referencing self or others

**DOI:** 10.18699/vjgb-24-105

**Published:** 2024-12

**Authors:** Q. Si, J. Tian, V.A. Savostyanov, D.A. Lebedkin, A.V. Bocharov, A.N. Savostyanov

**Affiliations:** Novosibirsk State University, Novosibirsk, Russia; Novosibirsk State University, Novosibirsk, Russia; Novosibirsk State University, Novosibirsk, Russia Scientific Research Institute of Neurosciences and Medicine, Novosibirsk, Russia; Novosibirsk State University, Novosibirsk, Russia Institute of Cytology and Genetics of the Siberian Branch of the Russian Academy of Sciences, Novosibirsk, Russia; Novosibirsk State University, Novosibirsk, Russia Tomsk State University, Tomsk, Russia; Novosibirsk State University, Novosibirsk, Russia Institute of Cytology and Genetics of the Siberian Branch of the Russian Academy of Sciences, Novosibirsk, Russia Scientific Research Institute of Neurosciences and Medicine, Novosibirsk, Russia

**Keywords:** neurocomputing technologies, hardware-software module, data processing methods, self-referential processes, resting-state EEG, default-mode network, interethnic differences, collectivism, нейровычислительные технологии, программно-аппаратный модуль, методы обработки данных, самоотнесенные процессы, ЭЭГ покоя, дефолт-система мозга, межнациональные различия, коллективизм

## Abstract

Neurocomputing technology is a field of interdisciplinary research and development widely applied in modern digital medicine. One of the problems of neuroimaging technology is the creation of methods for studying human brain activity in socially oriented conditions by using modern information approaches. The aim of this study is to develop a methodology for collecting and processing psychophysiological data, which makes it possible to estimate the functional states of the human brain associated with the attribution of external information to oneself or other people. Self-reference is a person’s subjective assessment of information coming from the external environment as related to himself/herself. Assigning information to other people or inanimate objects is evaluating information as a message about someone else or about things. In modern neurophysiology, two approaches to the study of self-referential processing have been developed: (1) recording brain activity at rest, then questioning the participant for self-reported thoughts; (2) recording brain activity induced by self-assigned stimuli. In the presented paper, a technology was tested that combines registration and analysis of EEG with viewing facial video recordings. The novelty of our approach is the use of video recordings obtained in the first stage of the survey to induce resting states associated with recognition of information about different subjects in later stages of the survey. We have developed a software and hardware module, i. e. a set of related programs and procedures for their application consisting of blocks that allow for a full cycle of registration and processing of psychological and neurophysiological data. Using this module, brain electrical activity (EEG) indicators reflecting individual characteristics of recognition of information related to oneself and other people were compared between groups of 30 Chinese (14 men and 16 women, average age 23.2 ± 0.4 years) and 32 Russian (15 men, 17 women, average age 22.1 ± 0.4 years) participants. We tested the hypothesis that differences in brain activity in functional rest intervals between Chinese and Russian participants depend on their psychological differences in collectivism scores. It was revealed that brain functional activity depends on the subject relevance of the facial video that the participants viewed between resting-state intervals. Interethnic differences were observed in the activity of the anterior and parietal hubs of the default-mode network and depended on the subject attribution of information. In Chinese, but not Russian, participants significant positive correlations were revealed between the level of collectivism and spectral density in the anterior hub of the default-mode network in all experimental conditions for a wide range of frequencies. The developed software and hardware module is included in an integrated digital platform for conducting research in the field of systems biology and digital medicine

## Abstract (ru)

Нейровычислительные технологии – область междисциплинарных исследований и разработок, которая находит широкое применение в современной цифровой медицине. Одна из задач нейровычислительных технологий состоит в создании методик изучения мозговой активности человека в условиях социально-ориентированной деятельности при помощи современных информационных подходов. Цель предлагаемого исследования – разработать методику сбора и обработки психофизиологических данных, позволяющую изучать функциональные состояния головного мозга человека, ассоциированные с отнесением внешней информации к самому субъекту или другим людям. Под самоотнесением (самореференцией) понимается субъективная оценка человеком поступающей из внешней среды информации как имеющей отношение к нему самому. Отнесение информации к другим людям или неодушевленным объектам – это оценка информации как сообщения о ком-то другом или о вещах. В современной нейрофизиологии сложились два подхода к исследованию самореференции: 1 – регистрация мозговой активности в условиях покоя с последующим опросом участника на предмет выявления самоотнесенных мыслей; 2 – регистрация мозговой активности, вызванной самоотнесенными стимулами. В представленной работе была апробирована технология, сочетающая регистрацию и анализ ЭЭГ с просмотром видеозаписей изображений лица самого испытуемого или незнакомого ему человека. Новизна нашего подхода состоит в использовании видеозаписей человеческого лица, полученных на первом этапе обследования, для индукции состояний покоя, ассоциированных с распознаванием информации о разных субъектах, на более поздних этапах обследования. Нами был разработан программно-аппаратный модуль, т. е. комплект связанных друг с другом программ и процедур их применения, состоящий из блоков, позволяющих проводить полный цикл регистрации и обработки психологических и нейрофизиологических данных. При помощи этого модуля показатели электрической активности головного мозга (ЭЭГ), отражающие индивидуальные особенности распознавания информации, отнесенной к самому себе и другим людям, были сопоставлены между группами из 30 китайских (14 мужчин и 16 женщин, средний возраст 23.2 ± 0.4 года) и 32 российских (15 мужчин, 17 женщин, средний возраст 22.1 ± 0.4 года) участников. Мы проверили гипотезу, что различия в мозговой активности в интервалах функционального покоя между китайскими и российскими участниками зависят от их психологических различий в показателях коллективизма. Было выявлено, что функциональная активность мозга зависит от субъектной отнесенности лицевого видео, которое участники просматривали между интервалами покоя. Межнациональные различия наблюдались в активности переднего и заднего хабов дефолт-системы и зависели от субъектной отнесенности информации. У китайских, но не у российских участников выявлены достоверные положительные корреляции между уровнем коллективизма и спектральной плотностью в переднем хабе дефолт-системы во всех экспериментальных условиях для широкого ряда частотных диапазонов. Разработанный программно-аппаратный модуль включен в интегрированную цифровую платформу для проведения исследований в области системной биологии и цифровой медицины.

## Introduction

Neurocomputing technology is a technical field aimed at the development of methods for collection and computer analysis of neurophysiological data, which is widely used in digital medicine to create new approaches to diagnosis and therapy of diseases. The purpose of neurocomputing technologies is to develop programs and devices for obtaining information about the anatomo-functional organization of the nervous system in the norm and in pathologies.

The theory of reference was proposed in the works of logicians and linguists of the first half of the 20th century (for an overview, see Yakovleva, 2011). Information referencing is the evaluation of incoming information as being related to a particular object or subject. The term “self-reference” refers to the evaluation of an event as being related to the very subject perceiving information about that event (Northoff et al., 2005; Neff, McGehee, 2010). The term “self-reference” is fundamentally different in its content from the terms “reflection” (thinking about oneself) and “self-control” (controlling one’s actions), as it does not refer to behavior management or self-assessment, but to the domain of analyzing the incoming information from the external environment as relevant or irrelevant to oneself. To date, two fundamentally different approaches to the study of neurophysiological markers of subjective attribution of information have emerged. In the first approach, brain activity (recorded via EEG, MEG, or fMRI) is recorded in conditions of functional rest, i. e., without performing experimental tasks (Knyazev et al., 2012, 2016). After completing the recor- ding of brain activity, participants are surveyed about their focus on self-referential events. Another approach is to present participants with several sets of stimuli with unambiguous The theory of reference was proposed in the works of logicians and linguists of the first half of the 20th century (for an overview, see Yakovleva, 2011). Information referencing is the evaluation of incoming information as being related to a particular object or subject. The term “self-reference” refers to the evaluation of an event as being related to the very subject perceiving information about that event (Northoff et al., 2005; Neff, McGehee, 2010). The term “self-reference” is fundamentally different in its content from the terms “reflection” (thinking about oneself) and “self-control” (controlling one’s actions), as it does not refer to behavior management or self-assessment, but to the domain of analyzing the incoming information from the external environment as relevant or irrelevant to oneself. To date, two fundamentally different approaches to the study of neurophysiological markers of subjective attribution of information have emerged. In the first approach, brain activity (recorded via EEG, MEG, or fMRI) is recorded in conditions of functional rest, i. e., without performing experimental tasks (Knyazev et al., 2012, 2016). After completing the recor- ding of brain activity, participants are surveyed about their focus on self-referential events. Another approach is to present participants with several sets of stimuli with unambiguous

The goal of our study is to develop a new experimental model that combines both approaches described above to study the self-referential activity of the human brain, i. e., those neurophysiological processes that underlie the selfreference of information. In this model, the participant is presented with external information (viewing video images) about him/herself or another person versus observing an inanimate object. In the intervals between viewing the video images, the participant closes their eyes and does not receive external stimulation for some time. The proposed technology includes a technique for organizing data collection based on combining EEG recording with video recording of human faces (Savostyanov et al., 2022), a technique for preprocessing EEG data to clean the target signal from irrelevant noise (Delorme, Makeig, 2004), a technique for localizing the sources of brain signals on the cortical surface and searching for statistical relationships between neurophysiological activity and psychological characteristics of the survey participants (Pascual-Margui, 2002). In addition, our approach includes psychological testing to identify participants’ personality traits and severity of depression symptoms. Within the framework of the proposed article, we will test the created technology to search for neurophysiological differences caused by different attitudes toward the self in groups of Russian and Chinese students. We hypothesize that Russians are more inclined to individualistic definition of their own personality, whereas the Chinese are more characterized by collectivistic ways of self-definition. The developed methods and computer programs for data collection and processing, as well as the actual data collected in this study, are included as one of the modules of the integrated digital platform “Bioinformatics and Systems Computational Biology”, which is being developed at the Institute of Cytology and Genetics of the Siberian Branch of the Russian Academy of Sciences.

## Materials and methods

Software module for data collection and processing. We have created a software module for data collection and processing, which is included in the integrated digital platform “Bioinformatics and Systems Computational Biology” that is being developed at ICG SB RAS. The module consists of both software products developed by the staff of ICG SB RAS and software tools from open sources. In total, all the blocks of the module allow us to carry out a complete cycle of collection and processing of psychological and neurophysiological data, starting from preliminary inter- viewing of participants to obtain their consent to be examined, and ending with statistical processing of the obtained results. The list of programs included in the module is given in Table 1.

**Table 1.**
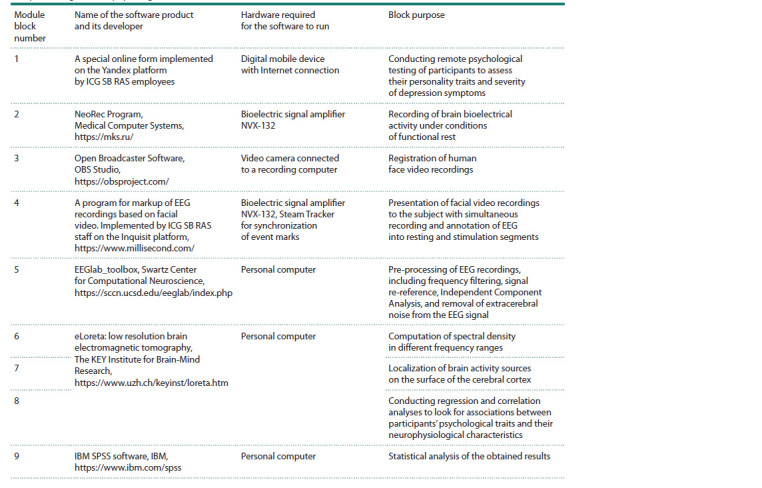
List of hardware and software blocks included in the module for registration and processing of neurophysiological data

Subjects. 30 undergraduate and PhD students from China (14 males and 16 females, mean age 23.2 ± 0.4 years) and 32 Russian undergraduate and PhD students (15 males, 17 females, mean age 22.1 ± 0.4 years), all studying at Novosibirsk State University, were invited. Before beginning the experiment, all participants completed a questionnaire that included questions about the presence of neurological or psychiatric diseases and alcohol or other psychoactive substance use. In addition, all participants gave informed consent to undergo the experimental examination in accordance with the Helsinki Declaration on Biomedical Ethics. The experimental protocol was approved by the ethical committee of the Scientific Research Institute of Neurosciences and Medicine.

Psychological evaluation. Participants filled out psychological questionnaires for trait-dependent and state-dependent anxiety (STAI: State-Trait Anxiety Inventory, Spielberger et al., 1970; Russian-language adaptation by Khanin, 1976), a questionnaire to assess the severity of depression symptoms (BDI: Beck’s Depression Inventory, Beck et al., 1996), the Collective and Individual Self-Concept Test (SCS: Self Construal Scale, Singelis, 1994), and the Relationally-Interdependent Self-Construal (RISC: Relational-interdependent self-construal, Cross et al., 2000). The survey was conducted using a special Internet application developed on the Yandex platform. Russian participants filled out questionnaires in Russian; Chinese participants, in Chinese.

**Fig. 1.**
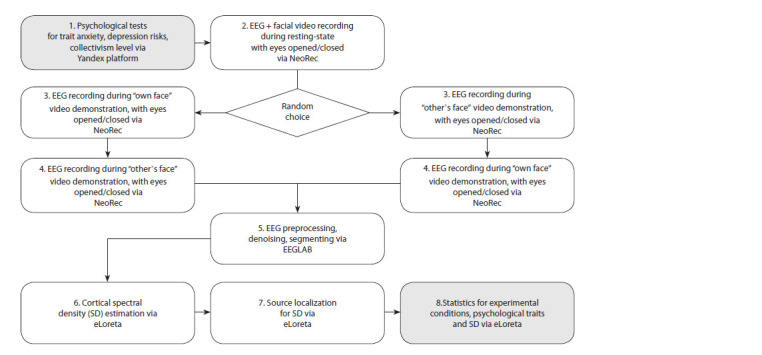
Flowchart of data collection and processing stages with references to the computer programs used in our study.

Experiment design, stages of data acquisition and processing. The experiment method and data processing steps are presented in the form of a flowchart in Figure 1. EEG was recorded in a sound- and light-isolated room. During the course of the experiment, three conditions were fulfilled. In the first experimental condition, EEG was recorded for 12 minutes without functional load (3 intervals of 2 minutes each with eyes closed and 3 intervals of 2 minutes each with eyes open). During the intervals when the subject opened their eyes, they saw a black screen of a computer monitor. During this period, the subject had a video image of their face recorded together with the EEG for all 12 minutes. The second and third conditions differed from the first in that in the second condition, with eyes open, the subject saw the video of their face obtained during the first condition, and in the third condition, they were presented with a video of an unfamiliar person’s face (always a male for a male subject and a female for a female subject). The order of the second and third task was randomly switched. For about half of the subjects, the second task came first, followed by the third task; for the other half, vice versa.

EEG recording. EEG was recorded using an NVX-132 amplifier, Russia. 128 EEG channels were arranged according to the international 5–5 % system with reference electrode Cz, ground electrode AFz, and additional channels for EOG and ECG. Bandwidth was set at 0.1–100 Hz, signal sampling frequency, at 1,000 Hz. The EEG recording was done using the NeoRec recorder software.

EEG preprocessing. Re-reference to the average was performed to remove artifacts of tonic scalp muscle tension. Oculomotor and other artifacts were removed from the EEG using Independent Component Analysis (ICA) from the EEGLAB software package version 14.1.2b for the MATLAB environment (Delorme, Makeig, 2004). ICA is a widely used data analysis technique that allows, among other things, to separate signal from noise. The EEG recordings were then divided into periods when the participant had their eyes closed and periods when their eyes were open. Further analysis was performed only for those EEG intervals that were recorded with closed eyes but were enclosed by the periods of the corresponding stimulus observation. Once these EEG segments were extracted, they were divided into two-second time intervals.

Brain activity sources localization on the cortex surface. Further analysis was performed using the eLoreta software package (Pascual-Margui, 2002). eLoreta is a mathematical model and a software product based on this model, aimed at solving the inverse problem of EEG, i. e. at reconstructing the sources of functional processes in the brain based on computer analysis of the distribution of electrical signals on the surface of the head. eLoreta allows localization of brain activity sources based on interpolation of data from numerous EEG electrodes.

For each two-second interval, spectral density values were calculated in the frequency bands of delta (2–4 Hz), theta (4–8 Hz), alpha-1 (8–10 Hz), alpha-2 (10–12 Hz), beta-1 (12–16 Hz), beta-2 (16–20 Hz), beta-3 (20–25 Hz), and gamma (25–35 Hz) rhythms. Then, for each participant, the total spectrum over the entire EEG trial inter- val was calculated separately for each of the three experimental conditions (150 to 170 two-second intervals were used for each participant). Spectra were computed independently for each of the 128 EEG channels included in the data processing. Source-level analysis of spectral den- sity comparisons between different conditions (“blank screen”, “own face”, and “other’s face”) was carried out in the eLoreta software. A 3,000 ms segment of the EEG recording with a sampling rate of 1,000 Hz after the onset of the block was used to calculate the spectral density

Statistical analysis of the results. Statistical analysis of the psychological assessment results was performed in the IBM SPSS software program. Comparisons were performed using one-way ANOVA with psychological traits as an independent variable, and intergroup factors “group” (Russian or Chinese), “gender” (male or female) and age as segregating variables.

Dependencies between experimental conditions and EEG metrics, and between psychological and ethnic characteristics and EEG metrics were assessed in the eLoreta package. The statistical significance of comparison results between different conditions was assessed using t-statistics for paired groups, with a randomization method of statistical nonparametric mapping (SnPM) that includes correction for multiple comparisons. The SnPM randomization method in eLoreta is based on a bootstrapping approach and is performed by multiple nonparametric permutation comparisons. A total of 5,000 randomizations were used to correct for multiple comparisons. Correlation analysis was performed to find the dependency of the spectral density on measures of personality traits and depression symptoms severity.

## Results 

Statistically significant results of the study and methods of their acquisition are presented in Table 2.

**Table 2.**
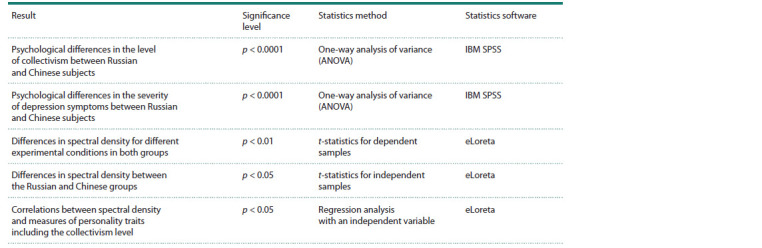
The main statistical results of the study, methods and software products used for obtaining them

## Results of psychological assessment

For the index of the anxiety trait according to the STAI test, the main effect of the “ethnicity” factor was not reliable (p > 0.3). A significant effect of the “gender” factor was found, F(1; 62) = 6.47, p = 0.014, η2 = 0.100, mean anxiety in women (30.6 ± 1.6) was higher than in men (24.8 ± 1.7). The BDI test revealed a statistically significant value of the “ethnicity” factor, F(1; 62) = 18.62, p < 0.0001, η2 = 0.243. The mean depression symptoms severity index was higher in the Chinese group (9.2 ± 1.1) than in the Russian group (2.8 ± 1.0).

**Fig. 2.**
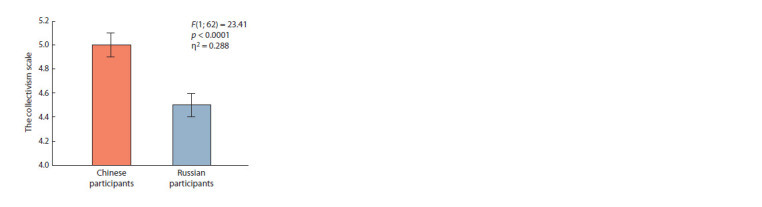
Differences between Chinese and Russian participants in terms of the collectivism score from the SCS questionnaire.

The RISC questionnaire revealed statistically significant differences between the ethnic groups, F(1; 62) = 7.27, p = 0.009, η2 = 0.111 in the importance of family values. The value of family was higher for Chinese participants (5.1 ± 0.2) than for Russian participants (4.3 ± 0.2). The SCS questionnaire also revealed a highly significant value for the “ethnicity” factor, F(1; 62) = 23.41, p < 0.0001, η2 = 0.288 for the collectivism indicator. For participants from the Chinese group, the collectivism index was higher (5.0 ± 0.1) than for participants from the Russian group (4.5 ± 0.1) (Fig. 2). There was a significant interaction between the factors “gender” and “nationality” for this indicator, F(1; 62) = 5.87, p = 0.019, η2 = 0.092. Russian (4.6 ± 0.1) and Chinese women (4.9 ± 0.1) did not differ significantly in this respect, whereas for Russian (4.3 ± 0.1) and Chinese (5.2 ± 0.1) men, the differences were more substantial.

## Results of eLoreta when comparing different experimental conditions for a generalized group (62 subjects, both Chinese and Russian participants) 

**Fig. 3.**
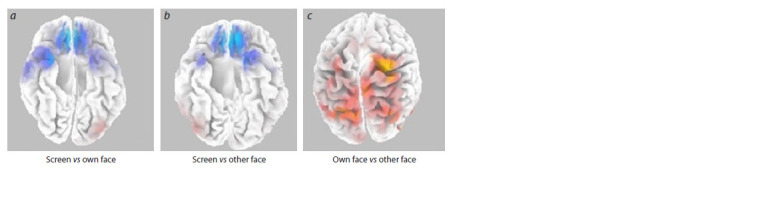
Comparison of spectral density in the alpha-2 (10–12 Hz) rhythm when comparing intervals with eyes closed between conditions (a) blank screen vs own face; (b) blank screen vs other’s face; (c) own face vs other’s face. The cortical regions in which spectral density is significantly higher for the “face” conditions than for the blank screen condition are marked in blue. Red color indicates cortical regions in which spectral density is significantly higher for the “own face” condition compared to the “other’s face” condition.

Using the eLoreta software package, spectral density metrics were compared for EEG intervals with eyes closed, which followed intervals of observing one’s own face, another person’s face, or a blank screen. It was found that spectral density in the frequency ranges of delta (2–4 Hz), alpha-2 (10–12 Hz), and gamma (25–35 Hz) rhythms was higher with eyes closed after observing one’s own face than with eyes closed after observing a blank screen. It should be specifically noted that muscle artifacts were removed from the EEG recordings using independent component analysis. According to Delorme and Makeig (2004), this method gives the ability to remove more than 80 % of all muscle noise. This suggests that the amplitude of electrical potentials in the delta and gamma bands was not simply due to surface tonic EMG. The statistically most reliable differences (p = 0.0036) were recorded for areas of the prefrontal cortex of both hemispheres (medial frontal area, 11 Brodmann’s area, and orbitofrontal cortex, 47 Brodmann’s area) in the range of the alpha-2 rhythm (Fig. 3a). Similar results were found when comparing the “other’s face” and “blank screen” conditions (Fig. 3b). Also, as in the first comparison, spectral density in the prefrontal cortex in the alpha-2 rhythm band is shown to be higher for the “other’s face” condition compared to the “blank screen” condition (p = 0.002). When comparing EEG intervals recorded after observing a stranger’s face, it was found that the spectral densities in the frequency bands of alpha-1 (8–10 Hz) and alpha-2 (10–12 Hz) rhythms in EEG intervals with eyes closed after observing a videotape of one’s own face were higher than in intervals with eyes closed after observing a stranger’s face. Significant differences in spectral density for these conditions (p = 0.0104) were found for the parietal cortex (superior parietal lobe, 7 Brodmann’s area, Fig. 3c).

## Results of eLoreta when comparing different experimental conditions for Chinese and Russian participants

**Fig. 4.**
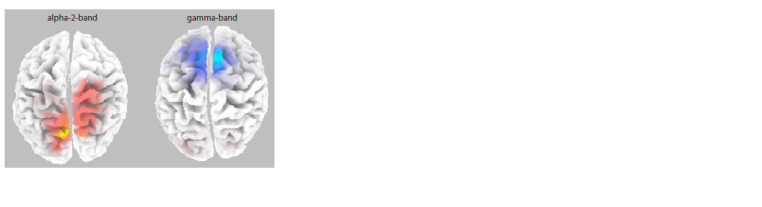
Comparison of spectral density in the alpha-2 (10–12 Hz) and gamma (25–35 Hz) bands when comparing Chinese and Russian groups for EEG intervals with eyes closed between the participants’ observation of their own face. This figure shows the superior surface of the cerebral cortex. Chinese participants are characterized by a greater, when compared to Russian participants, spectral density of the alpha-2 rhythm in the posterior (parietal and temporal) cortical regions (areas marked in red), whereas Russian participants were found to have significantly greater values of gamma rhythm spectral density in the medial frontal cortical regions (marked in blue).

Comparison of spectral density indices between the groups of Chinese and Russian subjects in intervals with eyes closed following the observation of a blank screen did not reveal any statistically significant intergroup differences. In this condition, both groups showed similar spectral density distributions in all cortical areas and all frequency bands. Cross-ethnic comparisons in the eyes-closed condition following observation of a videorecording of one’s face revealed significant differences in the alpha-2 and gamma rhythms (p = 0.044) (Fig. 4). Chinese participants in comparison with Russian participants showed increased spectral density in the alpha-2 band in the parietal and temporal cortex (38 Brodmann’s area), whereas Russian participants in comparison with Chinese participants showed increased spectral density in the medial frontal cortex in the gamma rhythm band (3, 4, and 6 Brodmann’s areas).

**Fig. 5.**
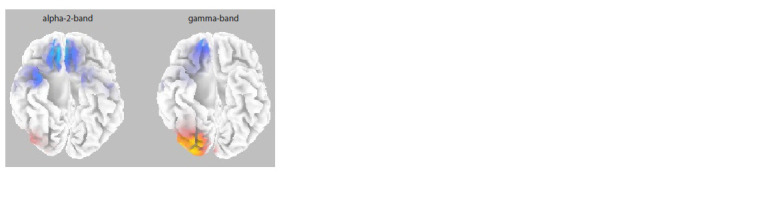
Comparison of spectral density in the alpha-2 (10–12 Hz) and gamma (25–35 Hz) bands when comparing the Chinese and Russian groups for EEG of “eyes closed” intervals between the intervals of the participants’ observation of a stranger’s face. This figure shows the inferior surface of the cerebral cortex The Chinese group is characterized by a greater, when compared to the Russian group, spectral density of gamma rhythms in the right inferior temporal cortex (areas marked in red), whereas the Russian group showed significantly greater values of spectral density of both alpha-2 and gamma rhythms in the prefrontal cortex (marked in blue).

Cross-ethnic comparisons in the eyes-closed condition between the observation of a stranger’s face video also revealed reliable differences in the ranges of alpha-2 and gamma rhythms (p = 0.0002), but they differed significantly from the results obtained for the own-face condition both in the topography of the effect and in the directionality of the cross-ethnic differences. Chinese participants in comparison to Russian participants showed significantly higher spectral density in the right inferior temporal cortical area (38 Brodmann’s area) in the gamma band, whereas Russian participants in comparison to Chinese participants showed higher spectral density values in both bands (alpha-2 and gamma rhythms) in the prefrontal cortical areas (medial frontal area, 11 Brodmann’s area and orbitofrontal cortex, 47 Brodmann’s area) (Fig. 5).

## Results of eLoreta in identifying the effects of psychological measures dependent on participants’ ethnicity and gender

The correlations between the SCS collectivism score for the combined group of Russian and Chinese subjects were statistically insignificant. There was no significance for the “blank screen” condition (p = 0.1954). For the “own face” (p = 0.0968) and “other’s face” (p = 0.0664) conditions for both groups, the p-levels were close to, but did not reach, a significant value.

**Fig. 6.**
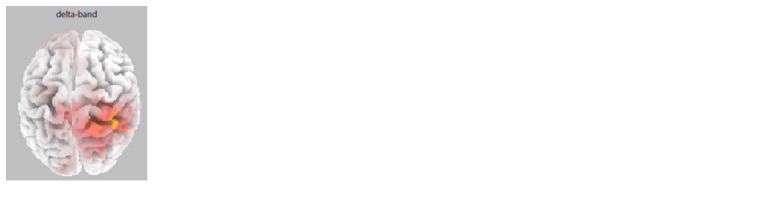
Correlations between the collectivism level and delta rhythm spectral density in the group of Russian participants in the “eyes closed” intervals following the observation of a stranger’s face. The cortical areas in which reliable positive correlations of the collectivism level with EEG spectral density measures were found are marked in red. The figure shows the convexital surface of the brain.

In the Russian sample for the collectivism index, no significant correlations were found for the “blank screen” or “own face” conditions. Significant correlations were found only for the spectral density in the delta band for the “other’s face” condition (p = 0.043) in the right temporal cortex (Brodmann’s area 22) (Fig. 6).

**Fig. 7.**
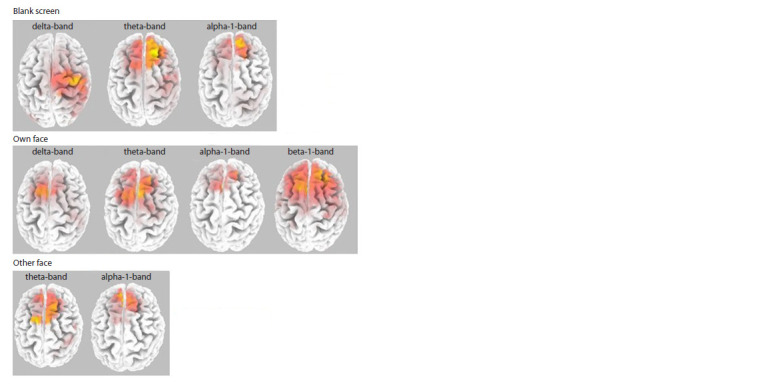
Correlations between the collectivism index and spectral density for the blank screen (first row), own face (second row), and other person’ face (third row) conditions in different frequency bands for Chinese participants. The cortical areas that showed positive correlations between the level of collectivism and spectral density on EEG are marked in red. The figure shows the convexital surface of the brain

In contrast to the Russian sample, for the Chinese participants, statistically significant correlations with the collectivism score were found for all three conditions (for “blank screen” p = 0.001, for “own face” p = 0.0032, for “other’s face” p = 0.0334). One can also notice that positive correlations with the collectivism score in the Chinese group were found for a wide range of delta, theta, alpha, and beta rhythms. These correlations are mainly found within the anterior cluster of the default- mode network (medial sections of the frontal and prefrontal cortex), and partially in the right parieto-temporal cortex (Fig. 7).

## Discussion

Development of a hardware-software module for data collection and analysis

The aim of this work was to create a neurocomputing technology and develop a hardware and software module for collecting and analyzing data to study brain processes underlying personal self-reference. We had previously proposed an approach that combines the analysis of resting EEG with the analysis of facial mimetic muscle activity recorded under the same conditions (Savostyanov et al., 2022). The main result of the new work is the demonstration of the possibility of using facial video recordings obtained at the initial stage of the experiment to initiate the participants’ processes of referencing information to themselves or others. Such data collection model is combined with well-known approaches for cleaning the EEG signal from noise (Delorme, Makeig, 2004) and localizing sources of brain activity on the surface of the cortex (Pascual-Margui, 2002).

One of the results of the study is the development of a hardware-software module that includes several sequentially connected blocks for experiment planning, data collection, preprocessing and analysis, as well as for intergroup statistical comparisons. In the future, this module can be used to conduct a wide range of neurophysiological studies, including the identification of markers of affective diseases such as depression, anxiety disorder, or autism spectrum disorders

Neurophysiological correlates of self-referential information processing

Researchers’ interest in studying the neurophysiological mechanisms of selfreferential information processing is driven, firstly, by the fundamental role of self-reference in the formation of human personality, and secondly, by the presence of a wide range of psychiatric diseases, the symptoms of which are various disorders in personal self-assessment (Bradley et al., 2016; Quevedo et al., 2018). In modern neurophysiology, there is a debate about the presence or absence of a specific anatomical substrate for self-referential processes in the brain (Northoff, Bermpohl, 2004; Northoff et al., 2005; Hu et al., 2016). The default-mode network, i. e., several interconnected cortical areas that show a decrease in the level of physiological activity when a person transitions from a resting state to performing cognitive tasks, is often considered as the main self-referential structure of the brain (Raichle, 2015; Knyazev et al., 2020, 2024).

The construction of a model of one’s own personality is significantly determined by the subject’s sociocultural specificity. In a classic study by Markus and Kitayama (1991), it was shown that representatives of Western (American) and Oriental (Japanese) cultures differ fundamentally in the criteria of the so-called “self-concept”, i. e. the way of selfidentification. Most Americans demonstrated individualistic personal attitudes, whereas collectivism was characteristic of the Japanese. In a cross-cultural study by G.G. Knyazev et al. (2012), a comparison of EEG correlates reflecting default-mode network activity at rest in representatives of Russian and Chinese (Taiwan) cultures was conducted. It was shown that most participants from Taiwan were characterized by dominance of the anterior (medial prefrontal cortex) hub of the default-mode network of the brain, whereas Russian participants showed dominance of the posterior (precuneus) part of this system (Knyazev et al., 2012). A hypothesis was proposed that interethnic differences in electrophysiological processes in the default-mode network may be caused by differences in self-concept according to the individualismcollectivism criterion, characteristic of representatives of Russian (predominantly individualistic) and Chinese (collectivistic) cultures. In our case, we experimentally tested the hypothesis of Knyazev et al. (2012) using data from the psychological questionnaires SCS and RISC.

Results of interethnic comparisons

The present study compared two samples of non-clinical subjects living in Russia at the time of the survey – Russian and Chinese. The examination included filling out psychological tests to identify the personality traits of the participants and the severity of their depression symptoms. The neurophysiological part of the examination consisted of EEG recording in three experimental conditions: (1) in the intervals between observation of a blank screen, (2) in the intervals between viewing a video of the participant’s own face, and (3) in the intervals between viewing a video of the face of a person unfamiliar to the participant.

Psychological comparisons showed that Russian and Chinese subjects did not differ in the anxiety trait (STAI test). As for the severity of depression (BDI test), it was found that Chinese subjects expressed depression symptoms more strongly than Russian participants. This difference can be explained by the fact that Chinese participants had been away from their home for a long time, whereas Russian participants were in more familiar conditions. In the measures of collectivism for both tests we used (RISC and SCS), highly reliable differences were found between Chinese and Russian participants. As expected, significantly higher collectivism scores were found for Chinese participants than for Russian participants.

Spectral density comparisons between the condition pairs “blank screen” vs “own face”, “blank screen” vs “other’s face”, “own face” vs “other’s face” for a generalized group of all participants regardless of their ethnicity and gender revealed statistically significant differences, predominantly in the alpha-2 rhythm range. Differences between neutral (blank screen) and both social (both own and other’s face) conditions were localized within the anterior hub of the default-mode network (medial prefrontal cortex). In both cases, the spectral density of the alpha rhythm was higher for the social than for the neutral condition. Differences between own and strangers’ faces were localized within the posterior hub of the default-mode network (medial parietal cortex) and were expressed in higher spectral density for own than for strangers’ faces.

Interethnic differences, without accounting for sex and psychological differences, were not detected in the EEG recorded in the intervals between blank screen observations, but were detected for the intervals between observations of both own and strangers’ faces. For the “own-face” condition, differences were found in the range of the alpha-2 rhythm in the posterior hub of the default-mode network (Chinese participants had higher spectral density than Russian participants), and in the range of the gamma rhythm in the anterior hub of the default-mode network (Russian participants had higher spectral density than Chinese participants). For the “foreign face” condition, a higher density of both alpha and gamma rhythm sources was detected in the anterior hub of the default-mode network in Russian participants, whereas for Chinese participants, a higher spectral density was detected in the temporal cortex. Thus, our result generally confirms the conclusion of G.G. Knyazev et al. (2012) about the presence of interethnic differences in the operation of the anterior and posterior hubs of the default-mode network.

In the group of Russian subjects, assessments of collectivism correlated with brain activity indices only for the “stranger’s face” condition. These correlations involved the posterior hub of the default-mode network. In contrast, in Chinese subjects, collectivism appeared to be a psychological metric for which multiple valid correlations were found for all three experimental conditions and several frequency ranges simultaneously. Most of the significant correlations in the Chinese group were found for brain structures from the anterior (medial frontal, medial prefrontal cortex) hub of the default-mode network. Thus, we confirm the hypothesis that the differences in default-mode network activity between Russian and Chinese subjects are mainly due to their differences in the collectivism index.

In general, thanks to the new experimental model proposed in this study, we were able to confirm G.G. Knyazev’s hypothesis that cross-cultural differences in default-mode net work activity between Chinese and Russian participants are associated with their differences in collectivism indicators

As a result of the study, we carried out the initial stage of development of a complex neurocomputing technology for collecting and analyzing psychological and physiological data, which allows to investigate the dynamics of processing self-referential information depending on the cultural features of the survey participants. The hardware-software module that we have developed is included in the integrated digital platform “Bioinformatics and Systems Computational Biology” being developed at ICG SB RAS under the budget project No. FWNR-2022-0020. It can be expected that the obtained approach will be further combined with the results of neurocomputer studies based on fMRI processing (Haxby et al., 2001) or with the data from psychogenetic studies. For example, for a portion of our subjects, data concerning their single-nucleotide polymorphisms in loci of brain neurotransmitter systems have been collected (Ivanov et al., 2022). Therefore, the results of psychological and neurophysiological studies can be compared with the genetic characteristics of the participants. In addition, convolutional neural networks using EEG metrics as input parameters can be used to classify participants into subgroups associated with different levels of stress (Fu et al., 2023).

## Conclusion

1. Brain electrical activity recorded during the intervals of functional rest following stimulation differs for conditions after presentation of neutral, self-referential, or otherreferential information to participants. This dependence is evident in measures of the spectral density of the alpha-2 rhythm in cortical regions that are part of the brain’s default-mode network.

2. Functional activity of the default-mode network in Chinese and Russian subjects differs in resting intervals following the observation of subject-referencing stimuli, but does not differ for intervals following the observation of a blank screen. Functional activity in the anterior and posterior hubs of the default-mode network depends significantly on the ethnicity of the participants

3. Functional activity in the anterior hub of the default-mode network is associated with collectivism in Chinese participants but not in Russian participants.

Limitations

1. During EEG recording, scalp EMG, which measures psychoemotional load, was not recorded. Although we performed the procedure of computing and applying the average reference, we can assume that the effects of personality traits and ethnicity in the gamma and beta bands are related not only to cerebral but also to muscular activity.

2. We chose standard rather than personalized frequency range boundaries, which may reduce the accuracy of identifying personalized EEG correlates of cognitive processes, especially for the alpha rhythm. Unfortunately, the software package we chose does not allow us to analyze spectral density in personalized ranges.

3. Although all female participants were interviewed before the experiment to establish the week of their menstrual cycle, we did not consider the psychoendocrinological factor of hormonal fluctuation in women when analyzing the EEG results, which may have reduced the accuracy of the findings.

We acknowledge all the limitations listed above and will strive to address them in future studies.

## Conflict of interest

The authors declare no conflict of interest.
